# Combination of Herpes Zoster and Muscular Atrophy

**Published:** 1882-06

**Authors:** 


### COMBINATION OF HERPES ZOSTER AND MUSCULAR ATROPHY.

Joffroy reported the following cases (*Archives de Physiologie*, 1882, No. 1). Case I.—Man, aged sixty-three; eruption of herpes zoster limited to right shoulder, and accompanied by severe pain. Three months later atrophy of the adductor pollicis, the interossei and the muscles of the hypothenar region was noticed. Faradic reaction almost lost, galvanic reaction heightened. Recovery after three months' application of galvanism.

Case II.—Man, aged forty; atrophy of the left deltoid occurred, with gradually increasing pain of left shoulder. Neuritis of the circumflex nerve was diagnosticated. After seven months' galvanic treatment almost complete recovery had occurred. Suddenly an eruption of herpes zoster appeared on the inner and posterior surface of the left forearm.

Joffroy thinks there was a probable connection between the zoster and the muscular atrophy. In the first case, according to his theory, an inflammation of sensory fibres of the brachial plexus extended to the adjoining motor fibres, and in the second case the process was reversed, the motor fibres being first affected and the sensory ones becoming secondarily involved.

Letulle (*ibid*) reports a case of what he terms "gangrenous zoster ophthalmicus" complicated with facial paralysis of the same side. [The description given of the eruption would seem to more nearly resemble the history of erysipelas.—*Tr.*] The case is as follows: a man aged fifty-one noticed a diffused and gradually increasing pain in the right half of the forehead. On the fifth day there appeared an eruption of broad, brownish vesicles, combined with bright redness and considerable œdema of the skin, extending over the right cheek and over part of the hairy scalp, nearly reaching the temporal region on both sides. At the same time the epidermis exfoliated in flakes of considerable size. During the eruption, which lasted eight days, the pain decreased and the sensibility appeared to be diminished. There was also bilateral submaxillary adenitis.

After removal of the crusts formed by the drying up of the vesicles, several gangrenous islets were observed among the freely suppurating patches, which rapidly healed by cicatrization.

On the twentieth day an incomplete paralysis of the right facial occurred, probably in consequence of taking cold.

Letulle believes that the various appearances in this case—erythema, œdema, desquamation and herpes zoster—were only stages of one and the same nerve-lesion—in this case a lesion of the ophthalmic branch of the fifth.

